# Thrombin action on astrocytes in the hindbrain of the rat disrupts glycemic and respiratory control

**DOI:** 10.1152/ajpregu.00033.2020

**Published:** 2020-04-22

**Authors:** Richard C. Rogers, Eileen M. Hasser, Gerlinda E. Hermann

**Affiliations:** ^1^Autonomic Neurosciences Laboratory, Pennington Biomedical Research Center, Baton Rouge, Louisiana; ^2^Biomedical Sciences, Dalton Cardiovascular Research Center, University of Missouri, Columbia, Missouri

**Keywords:** hyperglycemia, nucleus of the solitary tract, phrenic nerve activity, respiratory arrest, trauma

## Abstract

Severe trauma can produce a postinjury “metabolic self-destruction” characterized by catabolic metabolism and hyperglycemia. The severity of the hyperglycemia is highly correlated with posttrauma morbidity and mortality. Although no mechanism has been posited to connect severe trauma with a loss of autonomic control over metabolism, traumatic injury causes other failures of autonomic function, notably, gastric stasis and ulceration (“Cushing’s ulcer”), which has been connected with the generation of thrombin. Our previous studies established that proteinase-activated receptors (PAR1; “thrombin receptors”) located on astrocytes in the autonomically critical nucleus of the solitary tract (NST) can modulate gastric control circuit neurons to cause gastric stasis. Hindbrain astrocytes have also been implicated as important detectors of low glucose or glucose utilization. When activated, these astrocytes communicate with hindbrain catecholamine neurons that, in turn, trigger counterregulatory responses (CRR). There may be a convergence between the effects of thrombin to derange hindbrain gastrointestinal control and the hindbrain circuitry that initiates CRR to increase glycemia in reaction to critical hypoglycemia. Our results suggest that thrombin acts within the NST to increase glycemia through an astrocyte-dependent mechanism. Blockade of purinergic gliotransmission pathways interrupted the effect of thrombin to increase glycemia. Our studies also revealed that thrombin, acting in the NST, produced a rapid, dramatic, and potentially lethal suppression of respiratory rhythm that was also a function of purinergic gliotransmission. These results suggest that the critical connection between traumatic injury and a general collapse of autonomic regulation involves thrombin action on astrocytes.

## INTRODUCTION

Critical injuries, including head trauma, surgery-related bleeding, gunshot wounds, and burns can produce a posttraumatic “metabolic self-destruction” (26). This phenomenon is characterized by a catabolic profile including persistent hyperglycemia, functional insulin resistance, and greatly elevated metabolic fuel use. The association between severe trauma and hyperglycemia is clinically axiomatic (9, 26). The severity of the hyperglycemia is highly correlated with posttrauma morbidity and mortality (6, 13, 66). The outlook for injured patients is grave indeed when the metabolic consequences of their trauma cannot be successfully addressed (16). Insulin is used aggressively in the clinic to control posttrauma whole body glycemia and usually to good effect. However, the effects of insulin to induce central nervous system (CNS) cytoglucopenia in brain-injured patients produces its own morbidity, making strict insulin protocols for glycemic control controversial (16).

Under circumstances of “survivable” injury, this relationship between trauma and a stress-related increase in fuel availability assists in surmounting the traumatic event and subsequent healing (26). However, the extreme and prolonged hyperglycemia associated with the global metabolic collapse of severe trauma is not adaptive or helpful. Rather, it is better described as a pathophysiology in those individuals who would not have survived the initial insult had there not been timely clinical intervention. Despite the recognition of the association between trauma and metabolic pathophysiology, no detailed mechanism has been posited to connect severe trauma with metabolic control aside from a common citation of “CNS stress-related sympathetic activation” (16, 41).

However, traumatic injury also causes other failures of autonomic function. One of those, gastric stasis and ulceration (“Cushing’s ulcer”), has recently been connected with the generation of thrombin (31). As a protease, thrombin (produced as a consequence of bleeding or burn injury) acts on a unique class of G protein-coupled receptor, the protease-activated receptor (PAR). Four subtypes of receptors have been cloned (PAR1–4) (65); in the CNS, the PAR1 subtype is dominant (35). PARs possess their own tethered peptide ligand; a five-amino acid peptide. Serine proteases (e.g., thrombin) cleave a blocking peptide from the tethered ligand, allowing the short peptide to interact with the receptor within the extracellular loop to affect transmembrane signaling.

Several years ago, we hypothesized that the gastric stasis of traumatic injury occurred as a consequence of the activation by thrombin of PARs located on neurons in the autonomically critical nucleus of the solitary tract (NST) in the dorsal hindbrain. The NST is at the center of a homeostatic control nexus that receives vast quantities of visceral afferent data from cranial nerve afferents including the vagus. This region of the hindbrain is also outside the blood-brain barrier and is accessible to large molecules from the circulation. The NST parses this neural and chemical information to higher-order autonomic control networks to regulate gastrointestinal, metabolic, cardiorespiratory, endocrine, and behavioral functions (8, 30, 61, 63). To our initial surprise, we found that the PARs in the NST that regulate gastric function are located on astrocytes and not neurons (31). Our subsequent studies confirmed that activation of PARs on the NST astrocytes initiated glutamate “gliotransmission” onto NST neurons involved in autonomic control (74).

There may be a convergence between the effects of thrombin to derange hindbrain gastrointestinal control and the hindbrain circuitry that initiates counterregulatory responses (CRRs) to increase glycemia in reaction to critical hypoglycemia. Astrocytes in the hindbrain have been implicated as important detectors of low glucose or glucose utilization, and when activated, these astrocytes trigger CRR (37, 44, 45, 62, 64).

We tested the hypothesis that administration of thrombin to the floor of the fourth ventricle or directly into the NST could trigger increases in glycemia through the mediation of astrocytes. Preexposure of the fourth ventricle to SCH79797 (SCH; PAR1 antagonist) was used to evaluate whether thrombin effects were selective at PAR1 to evoke hyperglycemia. Fluorocitrate (FC; selective blocker of astrocyte metabotropic signaling) was used to evaluate astrocyte involvement in thrombin-triggered changes in glycemia. Pharmacological intervention in known gliotransmission pathways involving glutamate, purinergics, and likely transient receptor potential vanilloid (TRPV) channels was then performed. Preliminary studies revealed that thrombin not only caused increases in glycemia but also produced a rapid, dramatic, and potentially lethal suppression of respiratory rhythm. Both the glycemic and respiratory effects were mediated by purinergic gliotransmission.

## MATERIALS AND METHODS

All experimental procedures were conducted with the approval of either the Pennington Biomedical Research Center or the University of Missouri Institutional Animal Care and Use Committee and were performed according to the guidelines set forth by the National Institutes of Health. Long-Evans rats of either sex (31 females, 45 males; obtained from the Pennington Biomedical Research breeding colony) were used at Pennington in the first three experimental designs of this project. Male Sprague-Dawley rats (*n* = 24; obtained from Envigo, Indianapolis, IN) were used at the University of Missouri for the phrenic nerve activity experiments. Animal body weights were between 250 and 450 g, and ages ranged from 2 to 11 mo. At both centers, animals were housed in a temperature-controlled room under a 12:12-h light-dark cycle and provided water and food ad libitum.

### Fourth Ventricle Applications (Studies Conducted at Pennington Biomedical Research Center)

Animals were deeply anesthetized with thiobutabarbital [Inactin; 150 mg/kg ip, Sigma-Aldrich; long-term anesthesia with minimal interference on autonomic reflexes (10)]. With the use of an aseptic technique, a tracheal cannula (PE-240) was implanted, and the prepared animal was then secured in a stereotaxic frame. The floor of the fourth ventricle (4V) was exposed by removing the occipital skull plate and opening the foramen magnum; dura and arachnoid layers were retracted. Once all preparatory surgery was complete, systemic glucose levels were monitored via blood samples obtained by tail vein punctures. Glucose concentrations of the 3- to 4-μL blood droplets were determined with Freestyle Lite glucose test strips and glucometer (Abbott Diabetes Care, Alameda, CA). To ensure that baseline blood glucose levels were stable, samples were taken at 0, 30, and 60 min after all preparatory surgery was complete. At the 60-min point, each animal was exposed to one of the following 4V experimental conditions: *1*) control (sterile physiological saline, 10 μL; Henry Schein) followed 30 min later by thrombin from human plasma (4 U in 4 μL; cat. no. T4393, cell culture tested; Sigma-Aldrich, St. Louis, MO), “saline + thrombin”, *n* = 6 rats; *2*) SCH [potent and selective nonpeptide thrombin receptor antagonist; 1 nmol in 10 μL; Tocris Bio-Techne, Minneapolis, MN (1)] followed 30 min later by 4 U of thrombin, “SCH + thrombin”, *n* = 3 rats; *3*) fluorocitrate [FC; astrocytic metabotropic signal blocker, 5 nmol in 10 μL; Sigma-Aldrich) (17, 27, 43, 64, 70)] followed 30 min later by 4 U of thrombin, “FC + thrombin”, *n* = 7 rats; *4*) caffeine [nonselective adenosine receptor antagonist, 130 nmol in 6 μL; Sigma-Aldrich (59)] followed 30 min later by 4 U of thrombin; “caffeine + thrombin”, *n* = 6 rats; *5*) DPCPX [A1-specific adenosine antagonist; 8-cyclopentyl-1,3-dipropylxanthine; 2 nmol in 2 μL; Sigma Aldrich (40)] followed 30 min later by 4 U of thrombin; “DPCPX + thrombin”, *n* = 5 rats; *6*) dizocilpine [MK801; nonspecific *N*-methyl-d-aspartate receptor antagonist (NMDA), 18 nmol in 10 μL; Sigma Aldrich (12)] followed 30 min later by 4 U of thrombin; “MK801+thrombin”, *n* = 5 rats; *7*) [iodoresiniferatoxin (I-RTX); highly selective and extremely potent TRPV1 antagonist, previously identified as a potential gliotransmission antagonist, 4 pmol in 4 μL; Tocris Bio-Techne (68)] followed 30 min later by 4 U of thrombin; “I-RTX + thrombin”, *n* = 5 rats.

The 4-U thrombin dose used for the 4V challenge is at the low end of the dose range (4–20 U) used to model hemorrhagic stroke in the rat (32).

Tail vein blood glucose sampling occurred 30 min after blocker/antagonist pretreatment as well as 15, 30, 60, and 90 min after thrombin delivery. Baseline and peak levels of blood glucose were determined over this time course. Peak changes in blood glucose levels were expressed as percent changes relative to baseline for each individual animal; thus, each animal served as its own control. These normalized values of percent change in blood glucose levels for each group were averaged and subjected to a one-way analysis of variance followed by Dunnett’s post hoc tests for statistical significance (*P* < 0.05).

Preliminary studies suggested that thrombin applied to the 4V also produced immediate, dramatic, and long-lasting respiratory depression. To better quantify this effect, a microthermocouple probe (Omega MTSS, 0.25 mm diameter) was inserted into the opening of the tracheal cannula. Temperature differences in inspired versus expired air recorded by the probe were analyzed using a LabChart 7-PC system (AD Instruments). The first derivative of the raw temperature records was continuously plotted to provide a convenient record of respiratory rate. This was done using a rate meter in the LabChart software. Respiratory rates were monitored continuously throughout the experiment and charted on a minute-by-minute basis. Changes in breaths per minute were expressed as percent changes relative to baseline for each individual animal; thus, each animal served as its own control. These normalized values of percent change in respiration rate for each group were analyzed at 1, 5, 10, 15, and 30 min after 4V application of thrombin. Across these experimental groups, each of these time points was averaged and subjected to a one-way analysis of variance followed by Dunnett’s post hoc tests for statistical significance (*P* < 0.05).

### Artificial Respiration (Studies Conducted at Pennington Biomedical Research Center)

Respiratory depression and hypoxia have been reported to trigger hyperglycemia (38, 51, 57, 69, 72). With our observation of immediate and dramatic depression of respiration in the preliminary studies, we needed to determine if the hyperglycemia that ventricular thrombin provoked could be attributed solely to hypoxia. To address this question, a subgroup of rats supported by mechanical ventilation was added. Ventilatory rate was matched to the rat’s natural respiratory frequency, which varied from 50 to 60 breaths/min at a tidal volume of 6 mL/kg. That rate was then set on a Harvard model 683 rodent ventilator, where peak inspiratory pressure (PIP) was set to 15 cmH_2_O and positive end-expiratory pressure (PEEP) to 5 cmH_2_O (75).

A subset of 10 animals was surgically prepared as described above but with the addition of being artificially ventilated before and during the administration of 4 V saline and thrombin. Blood glucose levels were monitored as described previously.

### Direct Injections into the NST: Thrombin Effects on Glycemia (Studies Conducted at Pennington Biomedical Research Center)

Anesthesia, surgical installation of the tracheal tube, exposure of the hindbrain, determination of respiratory rate (for artificial ventilation), and preparations to record blood glucose were the same as for the studies described above. A micropipette with a 20-μm beveled tip was directed toward the medial NST (relative to the calamus scriptorius: 0.0 mm caudal, 0.4 mm lateral, and 0.4 mm ventral) via stereotaxic carrier. Thrombin (0.05 U in 50-nL volume) was nanoinjected from the pipette using pulses of air pressure (Pneumatic Picopump PV820, WPI). Injections into the NST were made under direct microscopic observation of the solution meniscus against a calibrated reticle (28).

Preliminary results suggested these thrombin injections produced an immediate and lethal suppression of respiratory rhythm. This observation and previous work suggesting that acute hypoxia can produce hyperglycemia (38, 69) required that we use artificial ventilation to maintain the animals for these nanoinjection studies. Therefore, mechanical ventilation was provided by the Harvard model 683 rodent ventilator, as described above.

Additional experimental groups of direct injections into the NST included the following: *1*) sterile physiological saline volume controls (50 nL), *n* = 5 rats; *2*) 4V saline (10 μL) followed 30 min later by nanoinjections of thrombin (0.05 U in 50 nL), *n* = 6 rats; *3*) 4V SCH (1 nmol in 10 μL) followed 30 min later by nanoinjections of thrombin, *n* = 4 rats; *4*) 4V FC (5 nmol in 10 μL) followed 30 min later by nanoinjections of thrombin, *n* = 6 rats; and *5*) 4V DPCPX (2 nmol in 2 μL) followed 30 min later by nanoinjections of thrombin, *n* = 8 rats. The dose used in direct NST nanoinjection while monitoring blood glucose levels was 0.05 U of thrombin in a 50-nL volume of sterile physiological saline.

To evaluate the tissue spread of injections, 50-nL volume injections of a solution of inert 0.04-μm red fluorescent plastic beads (FluoSpheres Invitrogen) was injected into the NST of two anesthetized rats. These rats were transcardially perfused with saline and 4% paraformaldehyde. Hindbrains were removed to a 30% sucrose solution overnight and then sectioned on a freezing microtome at 50 μm. Sections were then viewed on an upright epifluorescence microscope, and the fluorosphere injections sites were mapped.

### Direct Injections into the NST: Effective Doses of Thrombin to Disrupt CNS-Generated Phrenic Nerve Activity (Studies Done at Dalton Cardiovascular Research Center)

To validate the observation that thrombin in the hindbrain produced a dramatic suppression of neural respiratory rhythm we examined directly central respiratory output using a different approach in a different laboratory. A subset of animals was prepared for direct NST nanoinjection of thrombin with simultaneous monitoring of phrenic nerve activity, as performed previously (46, 47).

At the Dalton Cardiovascular Research Center at the University of Missouri, rats (*n* = 24) were anesthetized (isoflurane: 5%, induction; 2–3% maintenance, in 100% O_2_). Femoral venous and arterial catheters (PE-10 fused to PE-50, A-M Systems) were inserted to allow administration of drugs and routine measurement of arterial pressure and blood gases, respectively. A tracheotomy was performed, and rats were mechanically ventilated (683 Harvard Apparatus) with O_2_-enriched room air. Rats underwent bilateral section of the cervical vagus nerves to prevent entrainment of phrenic motor output with the ventilator. Arterial blood gases were measured (Osmetech OPTI CCA) periodically throughout the experiment, and tidal volume or respiratory range was adjusted for each animal as needed. Rectal temperature was monitored and maintained at ~38°C (Tele-Thermometer, Simpson Electrics). Note that because this protocol used isoflurane anesthesia, blood glucose measurements were not made (71).

The left phrenic nerve was isolated via a ventral cervical approach, placed on a bipolar silver recording electrode (0.005 in. bare, 0.007 in. coated, A-M Systems), and covered in silicone elastomer (Kwik-Cast, WPI), which was allowed to harden. The nerve was crushed distally, and the contralateral phrenic nerve was cut. Ground wires were inserted in muscle tissue near the electrodes and incision sites were closed. Nerve activity was amplified (×1,000), filtered (30–3,000 Hz, P511, Grass Technologies), rectified, and integrated using a root mean square converter (time constant = 100 ms). Background noise in the nerves was determined from the signal between bursts of activity, which we (46) have shown to be similar to that obtained after death. The recorded nerve activity minus noise was defined as phrenic nerve activity (PhrNA).

Rats were placed in a stereotaxic apparatus (Kopf Instruments). The brain stem was then exposed via partial occipital craniotomy as previously described (47). After surgical procedures were complete, isoflurane was reduced slowly, while anesthesia was converted gradually to intravenous thiobutabarbital (Inactin; 100 mg/kg, with 20 mg/kg supplements as needed). Animals were paralyzed using gallamine (8.3 mg/kg iv, 1–2 mg/h iv maintenance). Adequate plane of anesthesia was verified regularly by evaluation of the cardiovascular response to firm tail pinch (<5 mmHg increase in mean arterial pressure). Cardiorespiratory parameters were allowed to stabilize for at least 60 min before subsequent experimental manipulation. Ventilation with O_2_-enriched room air was established above apneic threshold by adjustment of tidal volume and breathing frequency. PhrNA, including phrenic frequency (PhrFreq), phrenic amplitude (PhrAmp), and minute phrenic nerve activity (MinPhrNA: PhrFreq × PhrAmp) was recorded continuously throughout the experiment.

A glass micropipette (1 or 2 barrels, depending on the protocol; ~10 μm/barrel) was advanced into the NST using dorsal brain stem surface landmarks. After the 60-min stabilization period, nanoinjections (30 nL) were made unilaterally into the NST (relative to calamus scriptorius: 0.0 mm caudal, 0.4 mm lateral, and 0.4 mm ventral). PhrNA was measured under control conditions, and animals were then exposed to combinations of the following: *1*) NST nanoinjections (30 nL, unilateral) of one of the following concentrations of thrombin reconstituted in artificial cerebrospinal fluid (aCSF): 0 U/μL (*n* = 6 rats), 0.005 U/μL (*n* = 4 rats), 0.01 U/μL (*n* = 7 rats), 0.02 U/μL (*n* = 7 rats), or 0.03 U/μL (*n* = 7 rats). The order of injections was varied to produce a balanced design. Note that the effective doses nanoinjected thrombin ranged from 0 to 0.0009 U (i.e., 30 nL of 0.3 U/μL = 0.0009 U total dose); *2*) nanoinjection (30 nL, 0.02 U/μL) thrombin outside the NST (relative to calamus scriptorius: 1.0 mm caudal, 2.0 mm lateral, and 0.5 mm ventral) to test for specificity of location (*n* = 4 rats); *3*) NST nanoinjection of thrombin (30 nL, 0.02 U/μL = 0.0006 U) 30 min after NST administration of FC (45 nL, 1 mM (*n* = 7 rats) or aCSF (30 nL, *n* = 6 rats).

Direct, unilateral nanoinjections of extremely low doses of thrombin resulted in robust but transient effects on PhrNA. Therefore, in keeping with using a minimum of animals, it was possible to expose each preparation to multiple doses of thrombin as well as pretreatments (control vs. FC) or nanoinjections outside the NST in a randomized fashion. Specific *n* numbers for each category are shown above.

### Data and Statistical Analysis

#### Blood glucose levels.

Tail vein blood glucose sampling occurred 30 min after blocker/antagonist pretreatment as well as 15, 30, 60, and 90 min after thrombin delivery. Baseline and peak levels of blood glucose were determined over this time course. Peak changes in blood glucose levels were expressed as percent changes relative to baseline for each individual animal; thus, each animal served as its own control. These normalized values of percent change in blood glucose levels for each group were averaged and subjected to a one-way analysis of variance followed by Dunnett’s post hoc tests for statistical significance (*P* < 0.05).

Blood glucose levels in artificially respirated animals after either 4V saline or thrombin were sampled as described above. Data were subjected to one-way analysis of variance followed by Bonferroni selected pairs posttest comparisons.

#### Respiratory rates.

Respiratory rates were monitored continuously throughout 4V thrombin experiments and charted on a minute-by-minute basis. Changes in breaths per minute were expressed as percent changes relative to baseline for each individual animal; thus, each animal served as its own control. These normalized values of percent change in respiration rate for each group were analyzed at 1, 5, 10, 15, and 30 min after 4V application of thrombin. Across these experimental groups, each of these time points were averaged and subjected to a one-way analysis of variance followed by Dunnett’s post hoc tests for statistical significance (*P* < 0.05).

#### Phrenic nerve activity (PhrNA).

PhrFreq and apnea duration data in response to different doses of thrombin nanoinjected directly into the NST were subjected to a one-way analysis of variance followed by Dunnett’s post hoc tests for statistical significance (*P* < 0.05). Pretreatment with FC to block the effects of thrombin on PhrFreq were compared with pretreatment with aCSF by use of a paired *t* test; *P* < 0.05 for statistical significance.

## RESULTS

### 4V Exposure to Thrombin and Effects on Blood Glucose

**Fig. 1.**
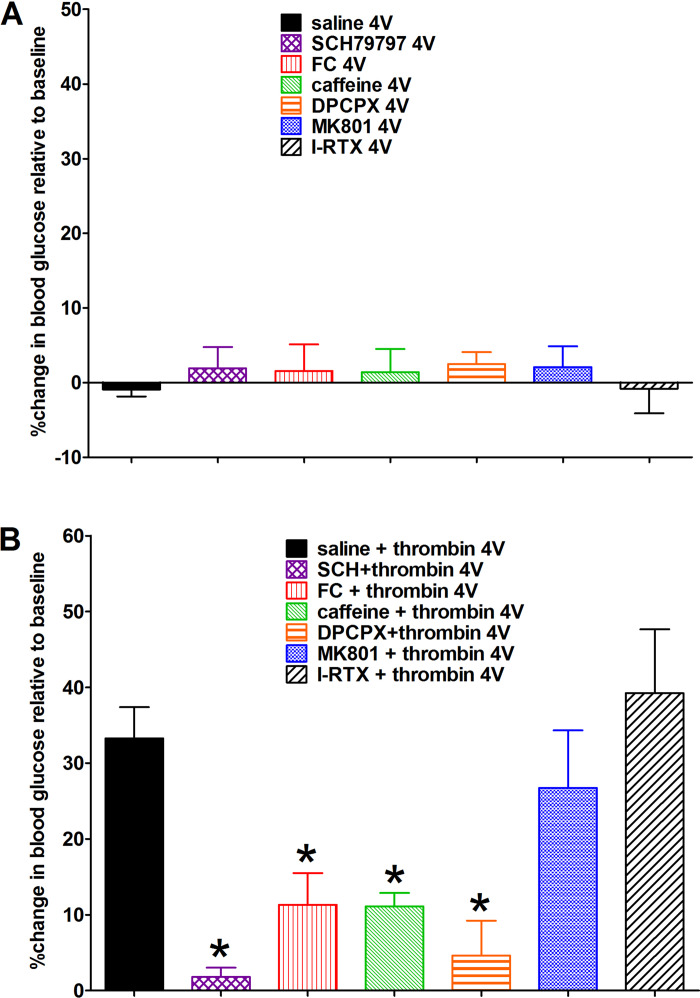
Percent changes in systemic blood glucose levels relative to baseline levels. *A*: fourth ventricular (4V) applications of saline or the antagonists [SCH79797 (SCH), fluorocitrate (FC), caffeine, 8-cyclopentyl-1,3-dipropylxanthine (DPCPX), dizocilpine (MK801), or iodoresiniferatoxin (I-RTX)], alone, had no effect on blood glucose; samples taken 30 min after 4V application. *B*: 4V administration of thrombin following saline pretreatment produced a significant increase in blood glucose, typically within 15–30 min of application, compared with control saline applications (in *A*). This thrombin effect on glycemia was blocked by pretreatment with FC (astrocyte signaling blocker), SCH [proteinase-activated receptor 1 (PAR1) antagonist)], caffeine (nonselective adenosine antagonist), and DPCPX (selective adenosine A1 antagonist). Neither *N*-methyl-d-aspartate receptor antagonist (NMDA) antagonist MK801 nor transient receptor potential cation channel V1 (TRPV1) antagonist I-RTX blocked the thrombin effect to increase glycemia (ANOVA, *F*_6,30_ = 7.2, *P* < 0.0001; Dunnett’s posttest against saline + thrombin, *P < 0.05).

4V applications of saline or the antagonists SCH, FC, caffeine, DPCPX, MK801, or I-RTX alone had no effect on blood glucose (Fig. 1*A*). 4V administration of thrombin following saline produced a significant increase in blood glucose, typically within 15–30 min of application compared with control saline applications. This thrombin effect on glycemia was blocked by pretreatment with FC (astrocyte signaling blocker), SCH (PAR1 receptor antagonist), caffeine (nonselective adenosine antagonist), and DPCPX (selective adenosine A1 antagonist). In contrast, neither the NMDA antagonist (MK801) nor the TRPV1 antagonist (I-RTX) blocked the thrombin effect to increase glycemia (Fig. 1*B*; ANOVA, *F*_6,30_ = 7.2, *P* < 0.0001; Dunnett’s posttest against saline + thrombin, *P < 0.05).

### Respiratory Depression After 4V Exposure to Thrombin

**Fig. 2.**
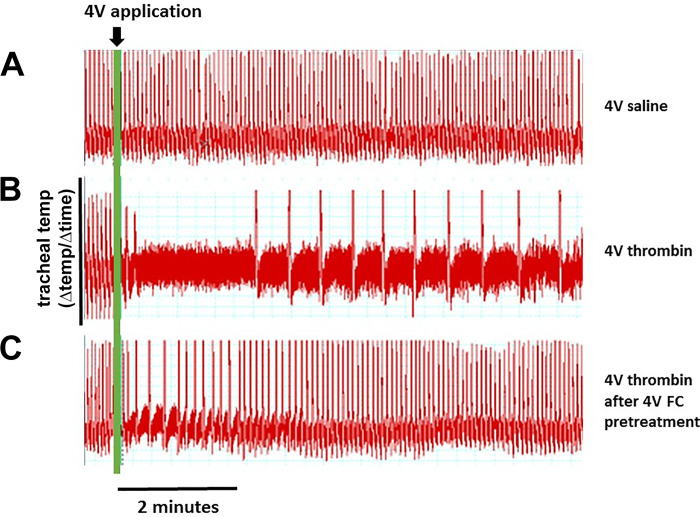
Respiratory rates were continuously monitored throughout the fourth ventricular (4V) thrombin experiments and charted minute by minute. A microthermocouple probe (Omega MTSS; 0.25 mm diameter) was inserted into the opening of the tracheal cannula. Temperature differences in inspired vs. expired air recorded by the probe were analyzed using a LabChart 7-PC system (AD Instruments). The first derivative of the raw temperature records was continuously plotted to provide a record of respiratory rate that could be quantified for statistical analysis. *A*: ~8-min record of respiratory rate following 4V application of saline. *B*: 4V application of thrombin produced a rapid, dramatic, and long-lasting respiratory depression. *C*: pretreatment of hindbrain with 4V application of fluorocitrate (FC) suppressed thrombin effects to disrupt respiration.

**Fig. 3.**
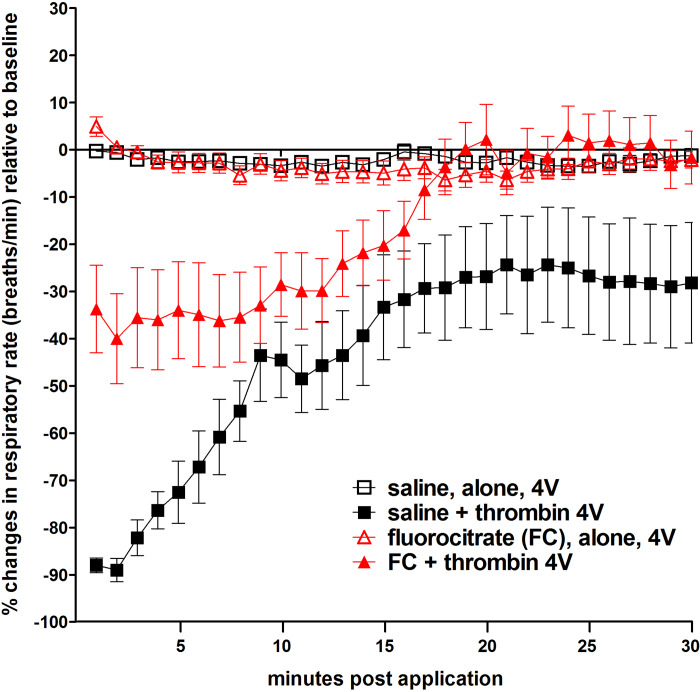
Plot of respiratory depression after fourth ventricular (4V) exposure to thrombin. Intraventricular application of thrombin produced a dramatic and nearly immediate suppression of respiration that persisted for more than 30 min after application (■). Neither saline (□) nor fluorocitrate (FC; red △), alone, had any demonstrable effects on respiration. However, pretreatment of the hindbrain with FC (red ▲) markedly altered the dynamics of this respiratory response to thrombin, in that the depression was not as large nor lasted as long.

**Fig. 4.**
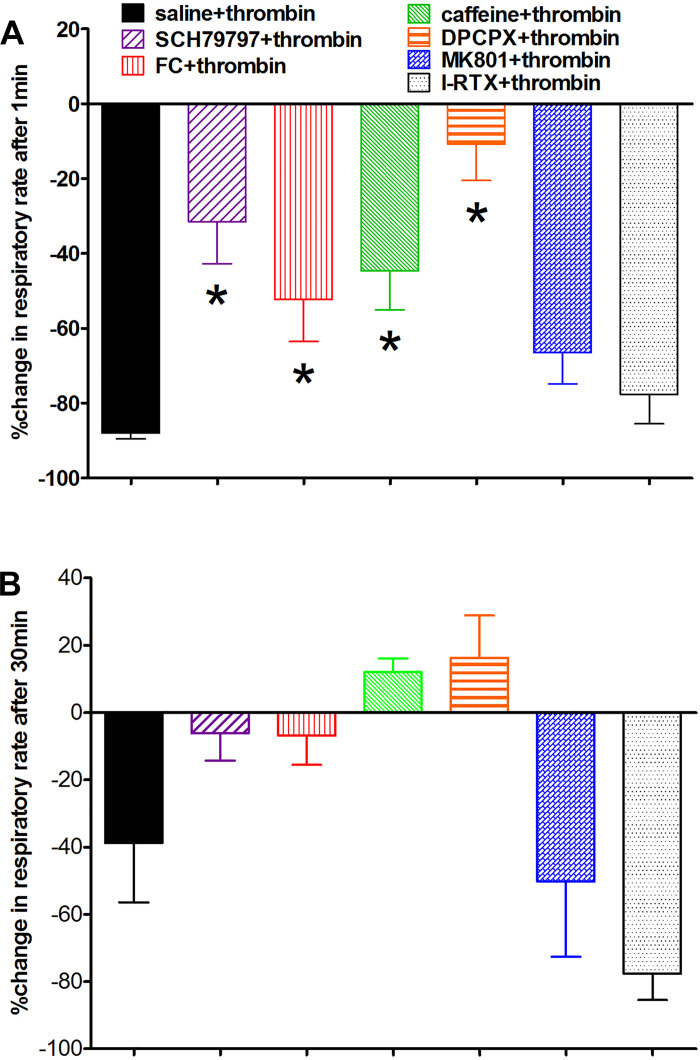
Percent change in respiratory rates, relative to baseline, after fourth ventricular (4V) exposure to thrombin. Effects of thrombin on respiration appeared to mirror the effects seen on blood glucose levels. *A*: pretreatment of the hindbrain with SCH79797, fluorocitrate (FC), caffeine, and 8-cyclopentyl-1,3-dipropylxanthine (DPCPX) blocked the respiratory depression effects of 4V thrombin normally observed within the first minute. Again, neither *N*-methyl-d-aspartate receptor antagonist (NMDA) antagonist dizocilpine (MK801) nor transient receptor potential cation channel V1 (TRPV1) antagonist iodoresiniferatoxin (I-RTX) blocked the thrombin effect on respiration (ANOVA, *F*_6,30_ = 7.3, *P* < 0.0001; Dunnett’s posttest against saline + thrombin, *P < 0.05). *B*: within 30 min after exposure to thrombin, respiratory rates of animals pretreated with SCH, FC, caffeine, or DCPCX had recovered to normal range.

We were surprised to see that thrombin applied to 4V produced a dramatic and nearly immediate suppression of respiration that persisted for ~30 min after application (Fig. 2). Pretreatment of the hindbrain with FC markedly altered the dynamics of this respiratory response to thrombin, in that the depression was not as large nor lasted as long (Figs. 2 and 3). Indeed, the effects of thrombin on respiration appeared to mirror the effects seen on blood glucose levels; that is, pretreatment with SCH, FC, caffeine, and DPCPX blocked the respiratory depression effects of thrombin. Again, neither the NMDA antagonist MK801 nor the TRPV1 antagonist I-RTX blocked the thrombin effect on respiration (Fig. 4; ANOVA, *F*_6,30_ = 7.3, *P* < 0.0001; Dunnett’s posttest against saline + thrombin, *P < 0.05).

### Effects of Artificial Respiration on Thrombin-Induced Hyperglycemia

**Fig. 5.**
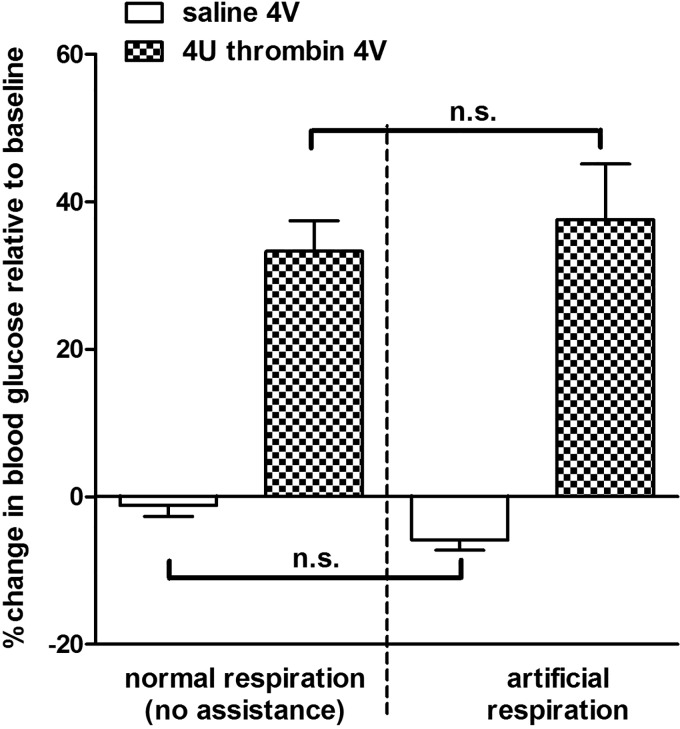
Effects of artificial respiration on thrombin induced hyperglycemia. This subset of experiments was designed to examine whether overriding hypoxia effects would also suppress hyperglycemia evoked by hindbrain thrombin. A subset of animals received artificial ventilation throughout the duration of the experiment. Blood glucose levels in artificially respirated animals after either fourth ventricular (4V) saline or thrombin were not significantly different from those we observed in nonventilated animals (ANOVA, *F*_3,27_ = 19.35, *P* < 0.0001; Bonferroni selected pairs posttest comparisons; n.s., not significant).

Our observations of the effect of thrombin on both blood glucose and respiration, together with reports in the literature that respiratory depression and hypoxia appear to trigger hyperglycemia (38, 51, 57, 69, 72), required us to examine whether overriding the hypoxia effects would also suppress the hyperglycemia evoked by hindbrain thrombin. This subset of animals received artificial ventilation, as detailed above, throughout the duration of the experiment. Blood glucose levels in artificially respirated animals after either 4V saline or thrombin were not significantly different from those we observed in the nonventilated animals (Fig. 5; ANOVA, *F*_3,27_ = 19.35, *P* < 0.0001; Bonferroni selected pairs posttest comparisons, not significant).

### Nanoinjection of Thrombin into the NST: Effects on Glycemia

**Fig. 6.**
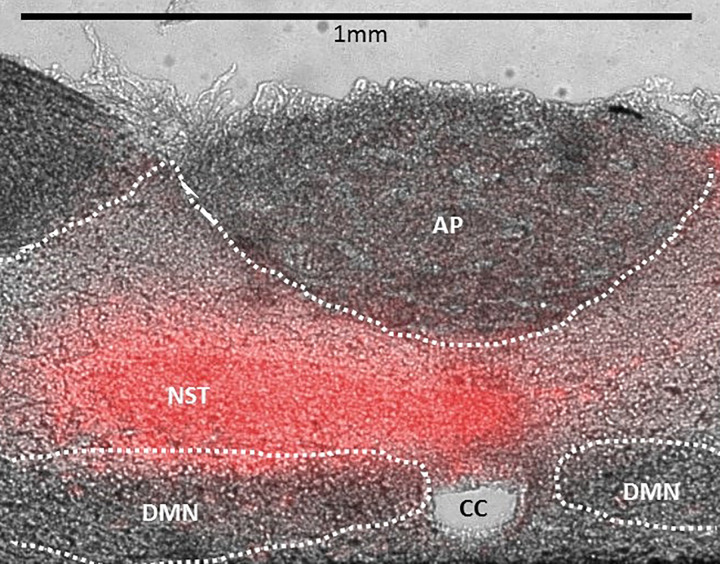
Histological verification of injection site and spread of injectate volume. Unilateral injections (50 nL) of fluorescent nanospheres demonstrated that this volume could fill the nucleus of the solitary tract (NST). AP, area postrema; CC, central canal; DMN, dorsal motor nucleus of the vagus; Scale bar, 1 mm.

**Fig. 7.**
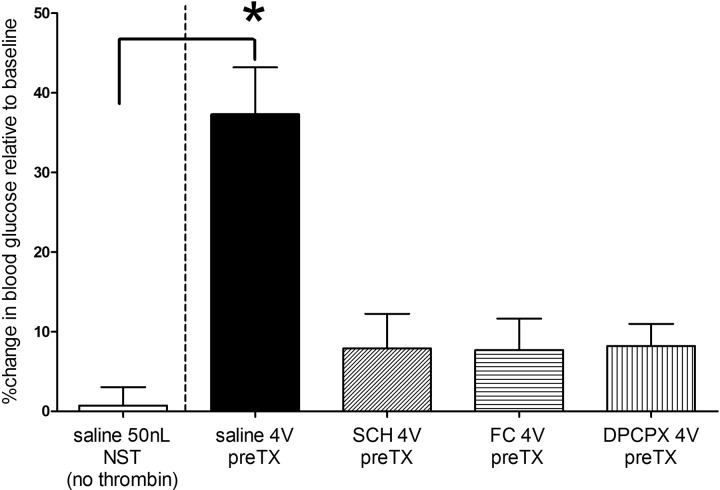
Effects of direct, unilateral injections (50 nL) into the nucleus of the solitary tract (NST) on blood glucose levels of artificially respirated rats. Mechanical ventilation combined with 50 nL of saline injections into the NST had no effect on blood glucose. Unilateral injections of thrombin (0.05 U in 50 nL) into the NST produced an increase in blood glucose similar to that observed with larger (i.e., 4 units) fourth ventricular (4V) applications (see Fig. 1). Similarly, the effects of NST thrombin on blood glucose levels were blocked by 4V pretreatment (pre-TX) with SCH79797 (SCH), fluorocitrate (FC), or 8-cyclopentyl-1,3-dipropylxanthine (DPCPX) (ANOVA, *F*_4,25_ = 12.32, *P* < 0.0001; Dunnett’s posttest comparisons against 50 nL saline + NST, *P < 0.05).

Fluorescent nanosphere injections revealed that 50-nL injection volumes filled the NST (Fig. 6). Mechanical ventilation combined with 50 nL of saline injections into the NST had no effect on blood glucose (Fig. 7). Unilateral injections of thrombin (0.05 U in 50 nL) into the NST produced an increase in blood glucose similar to that observed with the larger (i.e., 4-U) 4V applications. Likewise, the effects of NST thrombin on blood glucose levels were blocked by 4V pretreatment with SCH, FC, or DPCPX (ANOVA, *F*_4,25_ = 12.32, *P* < 0.0001; Dunnett’s posttest comparisons against saline 50 nL NST, *P < 0.05).

### Nanoinjection of Thrombin into the NST: Effects on Phrenic Nerve Activity

**Fig. 8.**
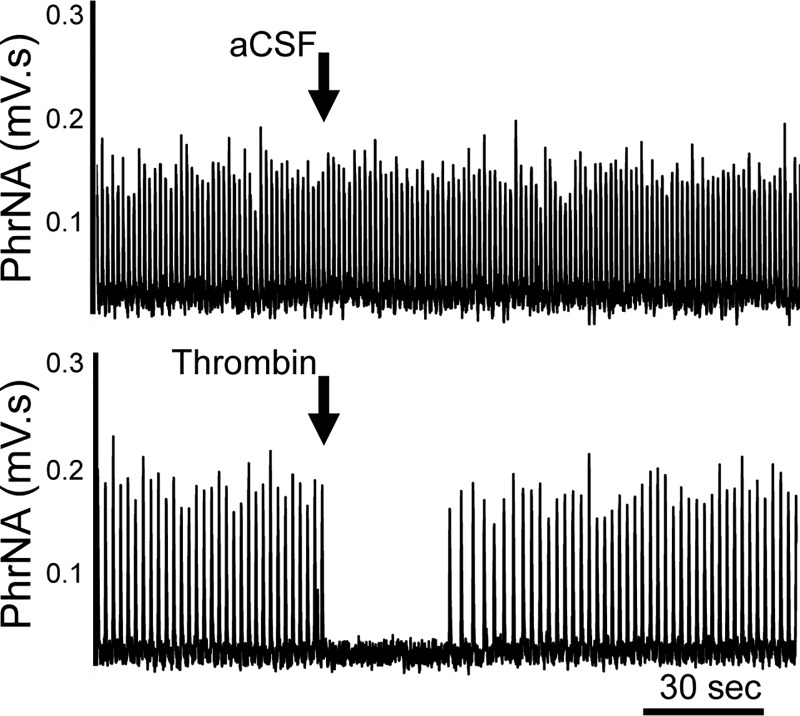
Representative examples of phrenic nerve activity (PhrNA) from two rats. *A*: unilateral nucleus of the solitary tract (NST) nanoinjection of artificial cerebrospinal fluid (aCSF; 30 nL) had little effect on neural respiration. *B*: in contrast, unilateral NST nanoinjection of thrombin (0.02 U/μL, 30 nL) inhibited PhrNA, due primarily to an effect on phrenic frequency.

**Fig. 9.**
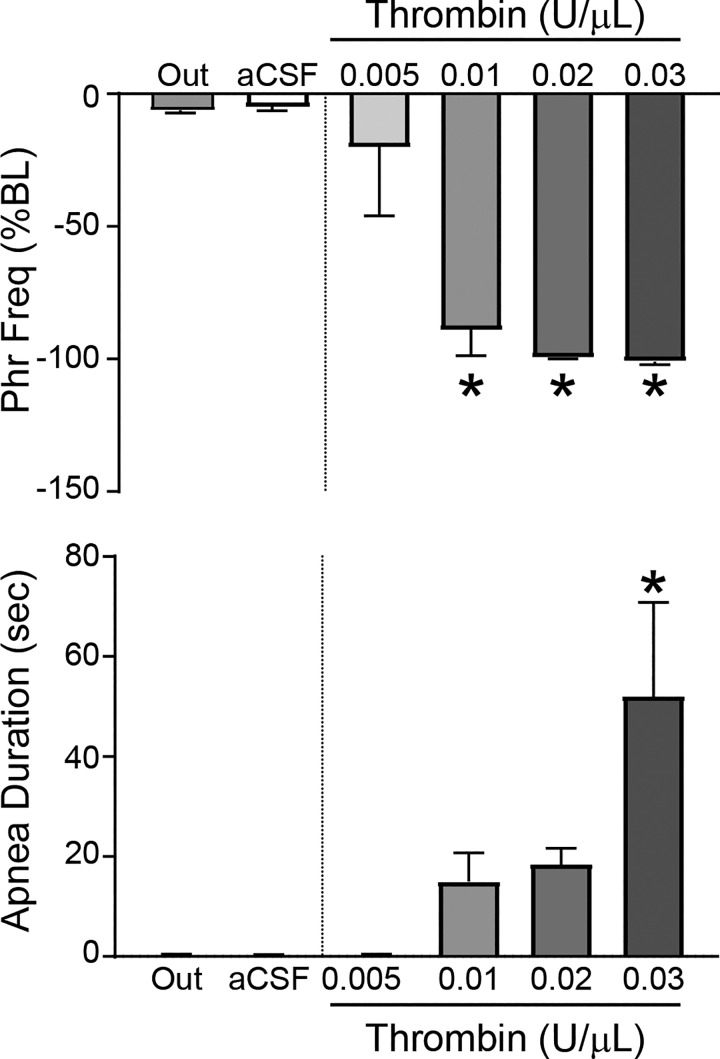
Thrombin in the nucleus of the solitary tract (NST) produces neural respiratory inhibition in a concentration-related fashion. Nanoinjection (30 nL) of increasing concentrations of thrombin into the NST decreased phrenic frequency (PhrFreq; *top*) and increased the duration of the neural apnea (*bottom*). ANOVA, *F*_5,28_ = 29.0, *P* < 0.0001; Dunnett’s posttest comparisons against artificial cerebrospinal fluid (aCSF), *P < 0.05. Nanoinjection of artificial cerebrospinal fluid (aCSF; *n* = 7) into the NST or nanoinjection of thrombin (0.02 U/μL, 30 nL) lateral to the NST (i.e., “Out”; *n* = 4) had minimal effects on phrenic nerve activity (not significantly different).

To verify that the respiratory depressant effects of thrombin were neurally mediated by actions in the NST, we measured the phrenic nerve activity response to unilateral nanoinjections of thrombin into the NST. A representative example demonstrates that nanoinjection of aCSF had minimal effects on PhrNA (Fig. 8*A*). In contrast, NST thrombin nanoinjection (Fig. 8*B*) produced an abrupt decrease in PhrNA, due primarily to a decrease in PhrFreq with little effect on PhrAmp. Group data indicate that NST nanoinjection of aCSF had little effect (Fig. 9), whereas thrombin injections into the NST induced a concentration-related decrease in PhrFreq (ANOVA, *F*_5,28_ = 29.0, *P* < 0.0001; Dunnett’s posttest comparisons against aCSF, *P < 0.05). In addition, the duration of the neural apnea in response to thrombin was concentration related. Nanoinjection of similar concentrations of thrombin outside the NST also had minimal effects that were not different from responses to NST aCSF, consistent with the concept that thrombin acts specifically within the NST to inhibit respiration.

**Fig. 10.**
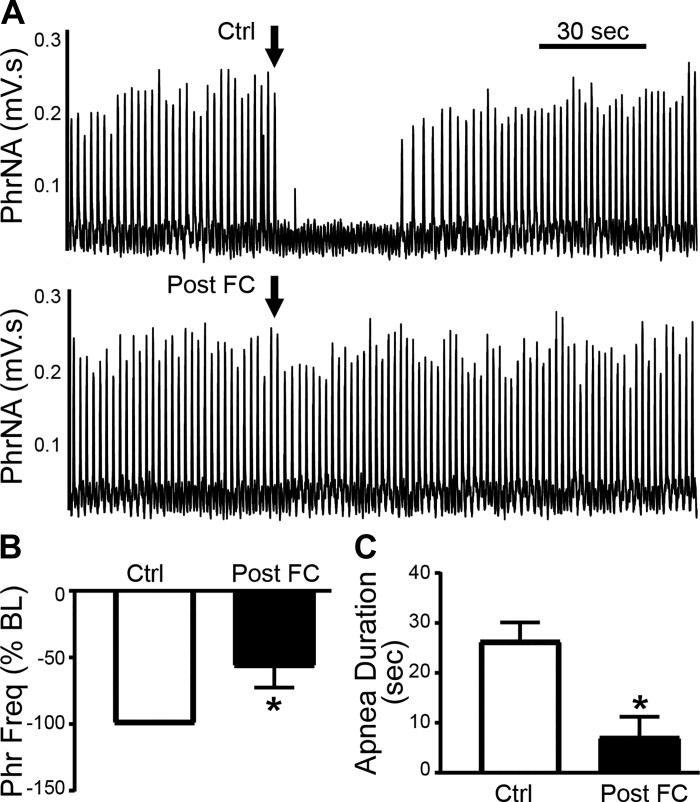
Astrocyte signaling blockade blunts the respiratory depressant effects of thrombin in the nucleus of the solitary tract (NST). *A, top*: representative recording from an individual rat showing that NST nanoinjection of thrombin (0.02 U/μL, 30 nL) under control (Ctrl) conditions of decreased phrenic frequency (PhrFreq). *Bottom*: in contrast, if pretreated with nanoinjection of fluorocitrate (FC) into the NST 30 min before thrombin nanoinjection (Post-FC), the decrease in PhrFreq was suppressed (*B*), as was the duration of neural apnea (*C*). *P < 0.05 vs. Ctrl.

To evaluate the role of astrocytes in the respiratory depressant effects of thrombin, we nanoinjected thrombin (0.02 U/μL, 30 nL) into the NST under control conditions and after astrocyte signaling blockade with FC (45 nL, 1 mM; *n* = 7). FC blunted the response to thrombin (Fig. 10*A*). Group data indicate that FC significantly reduced the thrombin effect on PhrFreq (*P* = 0.033, paired *t* test; Fig. 10*B*). The duration of phrenic apnea (Fig. 10*C*) was also significantly reduced by prior injection of FC (*P* = 0.012, paired *t* test), whereas prior aCSF administration had no effect (Ctrl: 20.8 ± 3.9 s vs. aCSF: 15.8 ± 3.5 s, *P* = 0.2, paired *t* test).

## DISCUSSION

Data from our studies demonstrate that the injections of thrombin in the hindbrain can provoke systemic hyperglycemia. Hindbrain thrombin also produces a dramatic depression of respiratory rhythm. Whereas acute hypoxia can produce hyperglycemia (38, 69), the current study demonstrated that artificial ventilation did not prevent the hyperglycemic response to thrombin. Therefore, hypoxia per se was not responsible for the increase in blood glucose levels observed after 4V exposure to thrombin. Pretreatment of the hindbrain with SCH (thrombin receptor antagonist), FC (astrocyte calcium signaling blocker), or adenosine receptor antagonists (caffeine and DPCPX) suppressed thrombin’s effects on hyperglycemia and respiratory depression. These results suggest that these physiological responses to thrombin in the hindbrain involve thrombin receptors, astrocytes, and purinergic gliotransmitters.

### Astrocytes and Neuronal Control

Long thought to only have a passive role in maintaining neuronal networks, it is now clear that astrocytes directly regulate neuronal excitability and synaptic plasticity. A single astrocyte may contact tens to hundreds of thousands of synapses and, along with presynaptic terminals and postsynaptic neurons, will form what has been termed the “tripartite synapse” (5, 11, 24, 25, 55). Astrocytes can be activated by neurotransmitters released from neuronal presynaptic terminals or gliotransmitters released by other astrocytes. Furthermore, astrocytes are sensitive to local, homeostatically regulated parameters such as blood gas tension and glucose availability (18, 20, 22, 23, 62, 64). Astrocytes are also stimulated by markers of immune activation such as cytokines (29, 52) and markers of traumatic injury such as thrombin (35, 74). All of these agents can act to increase astrocytic calcium levels (4, 7, 29, 31, 36, 39, 54, 56, 67). This increase in astrocytic calcium is coupled to a release of gliotransmitters (3, 24, 25, 55), such as glutamate, ATP, adenosine, and d-serine, among others (2, 7, 14, 19, 53). Astrocyte gliotransmission can potently affect the excitability of adjacent NST neuronal circuitry (18, 62, 74).

### Thrombin, Astrocytes, and Purinergic Control of Glycemia

Our earlier work on thrombin effects on NST function principally addressed the regulation of gastrointestinal function (31). The NST is the locus of vago-vagal circuit control of gastric motility and acid secretion. Traumatic injuries involving the production of thrombin are associated with clinical gastroparesis and erosive ulcers (Cushing’s ulcer) (42, 76). Thrombin or PAR1 agonists applied to this circuitry also causes gastroparesis (31). Follow-up neurophysiological and imaging studies show that these effects are, in turn, due to thrombin-induced glial release of glutamate, which modulates the activity of vagal afferents and NST neurons controlling gastric function (50, 74).

Thrombin-generating traumatic injury is also associated with a chronic hyperglycemia, whose magnitude is inversely correlated with injury survival (6, 9, 16, 26, 66). Counterregulatory hyperglycemic response to dangerously low plasma glucose is also controlled by circuitry in the hindbrain; specifically the NST and ventrolateral medulla (60). Our present studies demonstrated that thrombin applied to the floor of the 4V or injected directly into the NST causes a significant increase in blood glucose. This effect is clearly mediated by activation of PAR1 receptors on astrocytes, since 4V pretreatment with the specific PAR1 antagonist SCH blocks the effect, as does the astrocyte calcium-signaling inhibitor FC.

PAR1 activation of astrocytes by thrombin produces downstream effects to control glycemia through the release of adenosine. Caffeine, a nonselective adenosine antagonist, blocked the effects of thrombin to increase glycemia and depress respiration. The thrombin effect is probably mediated through the A1 adenosine receptor, since DPCPX, a selective A1 antagonist, completely blocked both the 4V and direct NST injection effects of thrombin. Surprisingly, blockade of NMDA or TRPV1 gliotransmission had no effect to inhibit thrombin-induced increases in plasma glucose. This is in contrast to the thrombin-astrocyte-glutamate gliotransmission responsible for gastroparesis (31, 74) and earlier reports concerning the involvement of TRPV1 channels in PAR suppression of respiratory control circuits (33). The involvement of purinergic gliotransmitter release in the thrombin induction of hyperglycemia is paralleled by our recent work showing that astrocytes activated by low-glucose challenge trigger counterregulatory responses through the release of purinergic agonists (62, 64).

While we were conducting studies of the effects of thrombin to alter glycemia, it was impossible to ignore the profound effect that thrombin had on respiratory rhythmogenesis. Even 4V application of thrombin (4 U) provoked an almost instantaneous suppression of respiration that required nearly 30 min to recover to near-normal patterns. Unilateral, direct injections of as little as 0.0003U (i.e., 30 nL of 0.01 U/μL) of thrombin into the NST significantly depressed PhrFreq activity and increased apnea duration. Pretreatment with unilateral, direct injections of FC suppressed this effect of thrombin. These data confirm that the respiratory depression is neurally mediated and support the concept that it involves astrocytes with the NST.

### Perspectives and Significance

Hindbrain astrocytes are emerging as important sources of chemosensory control over autonomic and autonomous functions such as glucose homeostasis and gastrointestinal, cardiovascular, and respiratory control (18, 20, 22, 23, 62, 64). Often, these functions are regulated by astrocyte release of the purines ATP and adenosine (18, 23, 58, 62, 64, 73). In particular, adenosine has been identified as a potent inhibitor of hindbrain respiratory rhythmogenesis (34). Suppression of purinergic gliotransmission seems to block these pathological outcomes of thrombin exposure, and this pharmacological approach might prove useful in the immediate management of the effects of trauma-produced thrombin effects on the brain.

Our observations of the effects of thrombin in the dorsal hindbrain to provoke pathological changes [first in gastrointestinal function (31), now in glycemic and respiratory control], reveal the profound and damaging effects that thrombin formation secondary to severe trauma can have on critical autonomic control circuitry. Although insulin therapy is commonly used to control whole body glycemia after trauma, the attendant CNS hypoglycemia that often results makes the aggressive use of insulin controversial, especially in the brain-injured patient. Insulin receptors on glial cells are likely to exert an important modulating influence on glucoregulatory circuits in the brain (21). It is not clear how insulin effects, hypoglycemia, and thrombin would, together, act on CRRs regulating astrocytes in the hindbrain. But each effect, individually, produces strong increases in intracellular calcium in target cells (15, 48, 49, 62). It remains to be seen whether these agents act on the same astrocytes controlling CRRs or on different astrocytes whose outputs converge on CRR-regulatory neurons.

## GRANTS

Funding support for this work came from National Institutes of Health Grants NS-60664, NS-096113, DK-108765, and HL-132836 and the John S. McIlhenny Professorship.

## DISCLOSURES

No conflicts of interest, financial or otherwise, are declared by the authors.

## AUTHOR CONTRIBUTIONS

R.C.R., E.M.H., and G.E.H. conceived and designed research; R.C.R., E.M.H., and G.E.H. performed experiments; R.C.R., E.M.H., and G.E.H. analyzed data; R.C.R., E.M.H., and G.E.H. interpreted results of experiments; R.C.R., E.M.H., and G.E.H. prepared figures; R.C.R., E.M.H., and G.E.H. drafted manuscript; R.C.R., E.M.H., and G.E.H. edited and revised manuscript; R.C.R., E.M.H., and G.E.H. approved final version of manuscript.

## References

[1] Ahn HS, Foster C, Boykow G, Stamford A, Manna M, Graziano M Inhibition of cellular action of thrombin by N3-cyclopropyl-7-[[4-(1-methylethyl)phenyl]methyl]-7H-pyrrolo[3, 2-f]quinazoline-1,3-diamine (SCH 79797), a nonpeptide thrombin receptor antagonist. Biochem Pharmacol 60: 1425–1434, 2000. doi:10.1016/S0006-2952(00)00460-3. 11020444

[2] Angulo MC, Kozlov AS, Charpak S, Audinat E Glutamate released from glial cells synchronizes neuronal activity in the hippocampus. J Neurosci 24: 6920–6927, 2004. doi:10.1523/JNEUROSCI.0473-04.2004. 15295027 PMC6729611

[3] Araque A, Carmignoto G, Haydon PG Dynamic signaling between astrocytes and neurons. Annu Rev Physiol 63: 795–813, 2001. doi:10.1146/annurev.physiol.63.1.795. 11181976

[4] Araque A, Martín ED, Perea G, Arellano JI, Buño W Synaptically released acetylcholine evokes Ca^2+^ elevations in astrocytes in hippocampal slices. J Neurosci 22: 2443–2450, 2002. doi:10.1523/JNEUROSCI.22-07-02443.2002. 11923408 PMC6758296

[5] Araque A, Parpura V, Sanzgiri RP, Haydon PG Tripartite synapses: glia, the unacknowledged partner. Trends Neurosci 22: 208–215, 1999. doi:10.1016/S0166-2236(98)01349-6. 10322493

[6] Ballian N, Rabiee A, Andersen DK, Elahi D, Gibson BR Glucose metabolism in burn patients: the role of insulin and other endocrine hormones. Burns 36: 599–605, 2010. doi:10.1016/j.burns.2009.11.008. 20074859

[7] Bezzi P, Carmignoto G, Pasti L, Vesce S, Rossi D, Rizzini BL, Pozzan T, Volterra A Prostaglandins stimulate calcium-dependent glutamate release in astrocytes. Nature 391: 281–285, 1998. doi:10.1038/34651. 9440691

[8] Blessing WW Anatomy of the lower brainstem. In: The Lower Brainstem and Bodily Homeostasis. Oxford, UK: Oxford University Press, 1997, p. 29–100.

[9] Bosarge PL, Kerby JD Stress-induced hyperglycemia: is it harmful following trauma? Adv Surg 47: 287–297, 2013. doi:10.1016/j.yasu.2013.03.002. 24298857

[10] Buelke-Sam J, Holson JF, Bazare JJ, Young JF Comparative stability of physiological parameters during sustained anesthesia in rats. Lab Anim Sci 28: 157–162, 1978. 642434

[11] Bushong EA, Martone ME, Jones YZ, Ellisman MH Protoplasmic astrocytes in CA1 stratum radiatum occupy separate anatomical domains. J Neurosci 22: 183–192, 2002. doi:10.1523/JNEUROSCI.22-01-00183.2002. 11756501 PMC6757596

[12] Campos CA, Ritter RC NMDA-type glutamate receptors participate in reduction of food intake following hindbrain melanocortin receptor activation. Am J Physiol Regul Integr Comp Physiol 308: R1–R9, 2015. doi:10.1152/ajpregu.00388.2014. 25394828 PMC4281681

[13] Cochran A, Scaife ER, Hansen KW, Downey EC Hyperglycemia and outcomes from pediatric traumatic brain injury. J Trauma 55: 1035–1038, 2003. doi:10.1097/01.TA.0000031175.96507.48. 14676647

[14] Coco S, Calegari F, Pravettoni E, Pozzi D, Taverna E, Rosa P, Matteoli M, Verderio C Storage and release of ATP from astrocytes in culture. J Biol Chem 278: 1354–1362, 2003. doi:10.1074/jbc.M209454200. 12414798

[15] Contreras-Ferrat A, Lavandero S, Jaimovich E, Klip A Calcium signaling in insulin action on striated muscle. Cell Calcium 56: 390–396, 2014. doi:10.1016/j.ceca.2014.08.012. 25224502

[16] Eakins J Blood glucose control in the trauma patient. J Diabetes Sci Technol 3: 1373–1376, 2009. doi:10.1177/193229680900300617. 20144391 PMC2787037

[17] Erlichman JS, Leiter JC Glia modulation of the extracellular milieu as a factor in central CO_2_ chemosensitivity and respiratory control. J Appl Physiol (1985) 108: 1803–1811, 2010. doi:10.1152/japplphysiol.01321.2009. 20110540 PMC2886686

[18] Erlichman JS, Leiter JC, Gourine AV ATP, glia and central respiratory control. Respir Physiol Neurobiol 173: 305–311, 2010. doi:10.1016/j.resp.2010.06.009. 20601205 PMC2946457

[19] Fellin T, Pascual O, Gobbo S, Pozzan T, Haydon PG, Carmignoto G Neuronal synchrony mediated by astrocytic glutamate through activation of extrasynaptic NMDA receptors. Neuron 43: 729–743, 2004. doi:10.1016/j.neuron.2004.08.011. 15339653

[20] Funk GD The “connexin” between astrocytes, ATP and central respiratory chemoreception. J Physiol 588: 4335–4337, 2010. doi:10.1113/jphysiol.2010.200196. 21078598 PMC3008839

[21] García-Cáceres C, Quarta C, Varela L, Gao Y, Gruber T, Legutko B, Jastroch M, Johansson P, Ninkovic J, Yi CX, Le Thuc O, Szigeti-Buck K, Cai W, Meyer CW, Pfluger PT, Fernandez AM, Luquet S, Woods SC, Torres-Alemán I, Kahn CR, Götz M, Horvath TL, Tschöp MH Astrocytic insulin signaling couples brain glucose uptake with nutrient availability. Cell 166: 867–880, 2016. doi:10.1016/j.cell.2016.07.028. 27518562 PMC8961449

[22] Gourine AV, Kasparov S Astrocytes as brain interoceptors. Exp Physiol 96: 411–416, 2011. doi:10.1113/expphysiol.2010.053165. 21257664

[23] Gourine AV, Kasymov V, Marina N, Tang F, Figueiredo MF, Lane S, Teschemacher AG, Spyer KM, Deisseroth K, Kasparov S Astrocytes control breathing through pH-dependent release of ATP. Science 329: 571–575, 2010. doi:10.1126/science.1190721. 20647426 PMC3160742

[24] Halassa MM, Fellin T, Takano H, Dong JH, Haydon PG Synaptic islands defined by the territory of a single astrocyte. J Neurosci 27: 6473–6477, 2007. doi:10.1523/JNEUROSCI.1419-07.2007. 17567808 PMC6672436

[25] Halassa MM, Haydon PG Integrated brain circuits: astrocytic networks modulate neuronal activity and behavior. Annu Rev Physiol 72: 335–355, 2010. doi:10.1146/annurev-physiol-021909-135843. 20148679 PMC3117429

[26] Hartl WH, Jauch KW Metabolic self-destruction in critically ill patients: origins, mechanisms and therapeutic principles. Nutrition 30: 261–267, 2014. doi:10.1016/j.nut.2013.07.019. 24369911

[27] Hassel B, Paulsen RE, Johnsen A, Fonnum F Selective inhibition of glial cell metabolism in vivo by fluorocitrate. Brain Res 576: 120–124, 1992. doi:10.1016/0006-8993(92)90616-H. 1515906

[28] Hermann GE, Nasse JS, Rogers RC Alpha-1 adrenergic input to solitary nucleus neurones: calcium oscillations, excitation and gastric reflex control. J Physiol 562: 553–568, 2005. doi:10.1113/jphysiol.2004.076919. 15539398 PMC1665513

[29] Hermann GE, Rogers RC TNF activates astrocytes and catecholaminergic neurons in the solitary nucleus: implications for autonomic control. Brain Res 1273: 72–82, 2009. doi:10.1016/j.brainres.2009.03.059. 19348788 PMC2693276

[30] Hermann GE, Rogers RC TNFalpha: a trigger of autonomic dysfunction. Neuroscientist 14: 53–67, 2008. doi:10.1177/1073858407305725. 17911224 PMC3951357

[31] Hermann GE, Van Meter MJ, Rood JC, Rogers RC Proteinase-activated receptors in the nucleus of the solitary tract: evidence for glial-neural interactions in autonomic control of the stomach. J Neurosci 29: 9292–9300, 2009. doi:10.1523/JNEUROSCI.6063-08.2009. 19625519 PMC2773000

[32] Hua Y, Keep RF, Hoff JT, Xi G Brain injury after intracerebral hemorrhage: the role of thrombin and iron. Stroke 38, Suppl: 759–762, 2007. doi:10.1161/01.STR.0000247868.97078.10. 17261733

[33] Huda R, Chang Z, Do J, McCrimmon DR, Martina M Activation of astrocytic PAR1 receptors in the rat nucleus of the solitary tract regulates breathing through modulation of presynaptic TRPV1. J Physiol 596: 497–513, 2018. doi:10.1113/JP275127. 29235097 PMC5792502

[34] Huxtable AG, Zwicker JD, Poon BY, Pagliardini S, Vrouwe SQ, Greer JJ, Funk GD Tripartite purinergic modulation of central respiratory networks during perinatal development: the influence of ATP, ectonucleotidases, and ATP metabolites. J Neurosci 29: 14713–14725, 2009. doi:10.1523/JNEUROSCI.2660-09.2009. 19940166 PMC6666021

[35] Junge CE, Lee CJ, Hubbard KB, Zhang Z, Olson JJ, Hepler JR, Brat DJ, Traynelis SF Protease-activated receptor-1 in human brain: localization and functional expression in astrocytes. Exp Neurol 188: 94–103, 2004. doi:10.1016/j.expneurol.2004.02.018. 15191806

[36] Kang J, Jiang L, Goldman SA, Nedergaard M Astrocyte-mediated potentiation of inhibitory synaptic transmission. Nat Neurosci 1: 683–692, 1998. doi:10.1038/3684. 10196584

[37] Klip A, Hawkins M Desperately seeking sugar: glial cells as hypoglycemia sensors. J Clin Invest 115: 3403–3405, 2005. doi:10.1172/JCI27208. 16322788 PMC1297271

[38] Koufakis T, Karras SN, Mustafa OG, Zebekakis P, Kotsa K The effects of high altitude on glucose homeostasis, metabolic control, and other diabetes-related parameters: from animal studies to real life. High Alt Med Biol 20: 1–11, 2019. doi:10.1089/ham.2018.0076. 30362832

[39] Lee CJ, Mannaioni G, Yuan H, Woo DH, Gingrich MB, Traynelis SF Astrocytic control of synaptic NMDA receptors. J Physiol 581: 1057–1081, 2007. doi:10.1113/jphysiol.2007.130377. 17412766 PMC2170820

[40] Lohse MJ, Klotz KN, Lindenborn-Fotinos J, Reddington M, Schwabe U, Olsson RA 8-Cyclopentyl-1,3-dipropylxanthine (DPCPX)—a selective high affinity antagonist radioligand for A1 adenosine receptors. Naunyn Schmiedebergs Arch Pharmacol 336: 204–210, 1987. doi:10.1007/BF00165806. 2825043

[41] Losser MR, Damoisel C, Payen D Bench-to-bedside review: glucose and stress conditions in the intensive care unit. Crit Care 14: 231, 2010. doi:10.1186/cc9100. 20727232 PMC2945096

[42] Lu WY, Rhoney DH, Boling WB, Johnson JD, Smith TC A review of stress ulcer prophylaxis in the neurosurgical intensive care unit. Neurosurgery 41: 416–426, 1997. doi:10.1097/00006123-199708000-00017. 9257310

[43] Martín ED, Fernández M, Perea G, Pascual O, Haydon PG, Araque A, Ceña V Adenosine released by astrocytes contributes to hypoxia-induced modulation of synaptic transmission. Glia 55: 36–45, 2007. doi:10.1002/glia.20431. 17004232

[44] Marty N, Dallaporta M, Foretz M, Emery M, Tarussio D, Bady I, Binnert C, Beermann F, Thorens B Regulation of glucagon secretion by glucose transporter type 2 (glut2) and astrocyte-dependent glucose sensors. J Clin Invest 115: 3545–3553, 2005. doi:10.1172/JCI26309. 16322792 PMC1297256

[45] Marty N, Dallaporta M, Thorens B Brain glucose sensing, counterregulation, and energy homeostasis. Physiology (Bethesda) 22: 241–251, 2007. doi:10.1152/physiol.00010.2007. 17699877

[46] Matott MP, Kline DD, Hasser EM Glial EAAT2 regulation of extracellular nTS glutamate critically controls neuronal activity and cardiorespiratory reflexes. J Physiol 595: 6045–6063, 2017. doi:10.1113/JP274620. 28677303 PMC5577520

[47] Matott MP, Ruyle BC, Hasser EM, Kline DD Excitatory amino acid transporters tonically restrain nTS synaptic and neuronal activity to modulate cardiorespiratory function. J Neurophysiol 115: 1691–1702, 2016. doi:10.1152/jn.01054.2015. 26719090 PMC4808102

[48] McDougal DH, Hermann GE, Rogers RC Astrocytes in the nucleus of the solitary tract are activated by low glucose or glucoprivation: evidence for glial involvement in glucose homeostasis. Front Neurosci 7: 249, 2013. doi:10.3389/fnins.2013.00249. 24391532 PMC3868892

[49] McDougal DH, Hermann GE, Rogers RC Vagal afferent stimulation activates astrocytes in the nucleus of the solitary tract via AMPA receptors: evidence of an atypical neural-glial interaction in the brainstem. J Neurosci 31: 14037–14045, 2011. doi:10.1523/JNEUROSCI.2855-11.2011. 21957265 PMC3445261

[50] McDougal DH, Viard E, Hermann GE, Rogers RC Astrocytes in the hindbrain detect glucoprivation and regulate gastric motility. Auton Neurosci 175: 61–69, 2013. doi:10.1016/j.autneu.2012.12.006. 23313342 PMC3951246

[51] Newhouse LP, Joyner MJ, Curry TB, Laurenti MC, Man CD, Cobelli C, Vella A, Limberg JK Three hours of intermittent hypoxia increases circulating glucose levels in healthy adults. Physiol Rep 5: e13106, 2017. doi:10.14814/phy2.13106. 28087818 PMC5256164

[52] Olmos G, Lladó J Tumor necrosis factor alpha: a link between neuroinflammation and excitotoxicity. Mediators Inflamm 2014: 861231, 2014. doi:10.1155/2014/861231. 24966471 PMC4055424

[53] Parpura V, Basarsky TA, Liu F, Jeftinija K, Jeftinija S, Haydon PG Glutamate-mediated astrocyte-neuron signalling. Nature 369: 744–747, 1994. doi:10.1038/369744a0. 7911978

[54] Pasti L, Volterra A, Pozzan T, Carmignoto G Intracellular calcium oscillations in astrocytes: a highly plastic, bidirectional form of communication between neurons and astrocytes in situ. J Neurosci 17: 7817–7830, 1997. doi:10.1523/JNEUROSCI.17-20-07817.1997. 9315902 PMC6793927

[55] Perea G, Navarrete M, Araque A Tripartite synapses: astrocytes process and control synaptic information. Trends Neurosci 32: 421–431, 2009. doi:10.1016/j.tins.2009.05.001. 19615761

[56] Porter JT, McCarthy KD Hippocampal astrocytes in situ respond to glutamate released from synaptic terminals. J Neurosci 16: 5073–5081, 1996. doi:10.1523/JNEUROSCI.16-16-05073.1996. 8756437 PMC6579292

[57] Rafacho A, Gonçalves-Neto LM, Ferreira FB, Protzek AO, Boschero AC, Nunes EA, Zoccal DB Glucose homoeostasis in rats exposed to acute intermittent hypoxia. Acta Physiol (Oxf) 209: 77–89, 2013. doi:10.1111/apha.12118. 23692825

[58] Rajani V, Zhang Y, Jalubula V, Rancic V, SheikhBahaei S, Zwicker JD, Pagliardini S, Dickson CT, Ballanyi K, Kasparov S, Gourine AV, Funk GD Release of ATP by pre-Botzinger complex astrocytes contributes to the hypoxic ventilatory response via a Ca^2+^-dependent P2Y1 receptor mechanism. J Physiol 596: 3245–3269, 2018. doi:10.1113/JP274727. 28678385 PMC6068109

[59] Ribeiro JA, Sebastião AM Caffeine and adenosine. J Alzheimers Dis 20, Suppl 1: S3–S15, 2010. doi:10.3233/JAD-2010-1379. 20164566

[60] Ritter S, Li AJ, Wang Q, Dinh TT Minireview: the value of looking backward: the essential role of the hindbrain in counterregulatory responses to glucose deficit. Endocrinology 152: 4019–4032, 2011. doi:10.1210/en.2010-1458. 21878511 PMC3444967

[61] Rogers RC, Hermann GE Brainstem control of gastric function. In: Physiology of the Gastrointestinal Tract (4th ed.), edited by Johnson LR Cambridge, MA: Elsevier Academic, 2012, p. 861–891.

[62] Rogers RC, McDougal DH, Ritter S, Qualls-Creekmore E, Hermann GE Response of catecholaminergic neurons in the mouse hindbrain to glucoprivic stimuli is astrocyte dependent. Am J Physiol Regul Integr Comp Physiol 315: R153–R164, 2018. doi:10.1152/ajpregu.00368.2017. 29590557 PMC6087883

[63] Rogers RC, McTigue DM, Hermann GE Vagovagal reflex control of digestion: afferent modulation by neural and “endoneurocrine” factors. Am J Physiol Gastrointest Liver Physiol 268: G1–G10, 1995. doi:10.1152/ajpgi.1995.268.1.G1. 7840189

[64] Rogers RC, Ritter S, Hermann GE Hindbrain cytoglucopenia-induced increases in systemic blood glucose levels by 2-deoxyglucose depend on intact astrocytes and adenosine release. Am J Physiol Regul Integr Comp Physiol 310: R1102–R1108, 2016. doi:10.1152/ajpregu.00493.2015. 27101298 PMC4935490

[65] Rohatgi T, Sedehizade F, Reymann KG, Reiser G Protease-activated receptors in neuronal development, neurodegeneration, and neuroprotection: thrombin as signaling molecule in the brain. Neuroscientist 10: 501–512, 2004. doi:10.1177/1073858404269955. 15534036

[66] Rovlias A, Kotsou S The influence of hyperglycemia on neurological outcome in patients with severe head injury. Neurosurgery 46: 335–342, 2000. doi:10.1097/00006123-200002000-00015. 10690722

[67] Scemes E, Giaume C Astrocyte calcium waves: what they are and what they do. Glia 54: 716–725, 2006. doi:10.1002/glia.20374. 17006900 PMC2605018

[68] Seabrook GR, Sutton KG, Jarolimek W, Hollingworth GJ, Teague S, Webb J, Clark N, Boyce S, Kerby J, Ali Z, Chou M, Middleton R, Kaczorowski G, Jones AB Functional properties of the high-affinity TRPV1 (VR1) vanilloid receptor antagonist (4-hydroxy-5-iodo-3-methoxyphenylacetate ester) iodo-resiniferatoxin. J Pharmacol Exp Ther 303: 1052–1060, 2002. doi:10.1124/jpet.102.040394. 12438527

[69] Strohl KP Diabetes and sleep apnea. Sleep 19, Suppl 10: S225–S228, 1996. doi:10.1093/sleep/19.suppl_10.S225. 9085517

[70] Swanson RA, Graham SH Fluorocitrate and fluoroacetate effects on astrocyte metabolism in vitro. Brain Res 664: 94–100, 1994. doi:10.1016/0006-8993(94)91958-5. 7895052

[71] Tanaka K, Kawano T, Tomino T, Kawano H, Okada T, Oshita S, Takahashi A, Nakaya Y Mechanisms of impaired glucose tolerance and insulin secretion during isoflurane anesthesia. Anesthesiology 111: 1044–1051, 2009. doi:10.1097/ALN.0b013e3181bbcb0d. 19809283

[72] Torrella M, Castells I, Gimenez-Perez G, Recasens A, Miquel M, Simó O, Barbeta E, Sampol G Intermittent hypoxia is an independent marker of poorer glycaemic control in patients with uncontrolled type 2 diabetes. Diabetes Metab 41: 312–318, 2015. doi:10.1016/j.diabet.2015.01.002. 25662841

[73] Turovsky E, Theparambil SM, Kasymov V, Deitmer JW, Del Arroyo AG, Ackland GL, Corneveaux JJ, Allen AN, Huentelman MJ, Kasparov S, Marina N, Gourine AV Mechanisms of CO_2_/H^+^ sensitivity of astrocytes. J Neurosci 36: 10750–10758, 2016. doi:10.1523/JNEUROSCI.1281-16.2016. 27798130 PMC5083006

[74] Vance KM, Rogers RC, Hermann GE PAR1-activated astrocytes in the nucleus of the solitary tract stimulate adjacent neurons via NMDA receptors. J Neurosci 35: 776–785, 2015. doi:10.1523/JNEUROSCI.3105-14.2015. 25589770 PMC4293422

[75] Vreugdenhil HA, Heijnen CJ, Plötz FB, Zijlstra J, Jansen NJ, Haitsma JJ, Lachmann B, van Vught AJ Mechanical ventilation of healthy rats suppresses peripheral immune function. Eur Respir J 23: 122–128, 2004. doi:10.1183/09031936.03.00035003. 14738243

[76] Young B, Ott L, Yingling B, McClain C Nutrition and brain injury. J Neurotrauma 9, Suppl 1: S375–S383, 1992. 1588628

